# Causes of death and mortality patterns in clozapine-treated patients: 17-year retrospective cohort study

**DOI:** 10.1192/bjo.2026.12069

**Published:** 2026-08-10

**Authors:** Mar Alonso-Garcia, David Seabright, Guluno Malik Achakzai, Amina Ali, Rajeev Krishnadas, Rudolf N. Cardinal, Christoper Jenkins, Celso Arango, Emilio Fernandez-Egea

**Affiliations:** Department of Psychiatry, Gregorio Marañón General University Hospital, Madrid, Spain; Cambridgeshire and Peterborough NHS Foundation Trust, Cambridge, UK; Department of Psychiatry, University of Cambridge, UK; IdiPAZ, School of Medicine, University Hospital La Paz, The Autonomous University of Madrid, CIBERSAM, Madrid, Spain

**Keywords:** Clozapine, mortality, schizophrenia, causes of death

## Abstract

**Background:**

Clozapine reduces overall mortality and suicide risk in schizophrenia, but the specific causes and clinical contexts of death among clozapine-treated patients remain less well characterised.

**Aims:**

We aimed to characterise causes of death in clozapine-treated patients within a defined UK catchment area over 17 years (2009–2025); compare features across predefined mortality clusters (suicide, expected, unexpected); compare unexpected death cases with a clozapine-treated comparison cohort alive in 2019 and examine temporal mortality patterns.

**Method:**

We conducted a retrospective, descriptive cohort study of all deaths among clozapine-treated patients within a UK mental health National Health Service Trust between 1 January 2009 and 31 December 2025. Deaths were classified into suicide, expected and non-intentional unexpected, using a previously established framework. Variables were compared across clusters and between unexpected death cases and a clozapine-treated comparison cohort alive in 2019.

**Results:**

Of 87 deaths, 11 (12.6%) were suicides, 29 (33.3%) were expected and 47 (54.0%) were non-intentional unexpected. Malignancy was the most common cause (21/87, 24.1%), followed by cardiovascular (13/87, 14.9%) and respiratory or infective causes (9/87, 10.3%). Age differed across clusters (*p* = 0.005), with suicides at younger ages. Compared with the 2019 cohort, patients who died unexpectedly were older (55.0 *v*. 48.6 years, *p* = 0.006) and more likely to smoke (75.0 *v*. 34.6%, *p* < 0.001). Annual mortality peaked in 2021 and was not fully explained by direct COVID-19 deaths.

**Conclusions:**

Mortality in clozapine-treated patients arises across diverse clinical contexts, with more than half classified as non-intentional unexpected. Findings support sustained physical health monitoring, closer attention to smoking and other modifiable risks, and continuity of care.

Individuals with schizophrenia continue to experience a markedly reduced life expectancy of approximately 15–20 years compared with the general population.^1^ Premature mortality in schizophrenia-spectrum disorders is primarily driven by cardiovascular disease and suicide, reflecting the combined impact of psychiatric illness, physical comorbidity, lifestyle factors, medication side effects and healthcare disparities.^2^ A better understanding of the cause of death would allow the implementation of more effective strategies.

Clozapine represents a unique case in the treatment of schizophrenia: it is often presented as associated with a deleterious adverse-effect profile due to the intensive monitoring requirements.^3^ Quite the opposite, clozapine is the only antipsychotic shown to reduce overall mortality^4–7^ and suicide risk^4^ in schizophrenia. Compared with other antipsychotics, clozapine has been associated with substantial reductions in all-cause and suicide-specific mortality, with reported relative risk reductions of approximately 30–40% for all-cause mortality^2,8^ and around 25% for suicide-specific mortality.^9,10^ Clozapine remains the only antipsychotic approved by the US Food and Drug Administration for the reduction of recurrent suicidal behaviour in patients with schizophrenia or schizoaffective disorder. Evidence from large national and registry-based studies consistently demonstrates a survival advantage among clozapine-treated patients, an effect thought to be largely attributable to its well-established reduction in suicidal behaviour. However, such datasets provide limited insight into the specific causes and clinical circumstances of death, particularly those that may be potentially preventable.

Causes of death among individuals with schizophrenia span a wide range of clinical contexts, from suicide to diverse physical-health-related causes, including both well-recognised and unexpected events.^6,11^ Clozapine-treated patients represent a clinically distinct subgroup within this broader population, characterised by treatment resistance, greater illness severity, and a high burden of physical comorbidity.^1,12^ Some deaths reflect natural ageing and the accumulation of physical comorbidity. However, a range of potentially preventable causes has been described, as clozapine treatment has been associated with cardiovascular disease, respiratory and infectious complications^11^ – particularly pneumonia^13,14^ – as well as malignancy. Recent population-based studies have refined estimates of cause-specific mortality among clozapine-treated patients. The Finnish FIN20 nationwide register study reported a particularly high burden of pneumonia and gastrointestinal hypomotility,^13^ while data from large East-Asian cohorts have confirmed an overall mortality benefit attributable to clozapine alongside differential risks for cardiovascular and infectious causes;^7^ pharmacovigilance evidence likewise indicates that respiratory infections, rather than haematological complications, account for the majority of fatal adverse drug reactions in this population.^15^ In addition, clozapine has been associated with rare but serious adverse reactions, including dose-related effects such as seizures^16^ or gastrointestinal hypomotility,^17^ and idiosyncratic or immune-mediated events such as agranulocytosis^18^ or myocarditis.^19,20^ A detailed characterisation of deaths occurring during clozapine treatment may help distinguish expected trajectories from potentially preventable events and inform targeted clinical monitoring strategies.

In a previous report from our catchment area, Cambridgeshire and Peterborough in the United Kingdom (UK), approximately half of deaths among clozapine-treated patients were classified as unexpected and potentially preventable.^11^ Building on this work, the present study extends the observation period to 17 years (2009–2025), spanning the COVID-19 pandemic and afterwards, thereby enabling examination of mortality patterns and contextual features observed during this unprecedented interval. Using the same classification framework and case-review approach previously described by Rose et al,^11^ this study sought to characterise causes of death among clozapine-treated patients and to examine mortality patterns across predefined clusters, with particular attention to non-intentional unexpected deaths as potentially preventable events.

## Method

### Study design and setting

This study was a retrospective descriptive analysis of mortality among patients prescribed clozapine, conducted within Cambridgeshire and Peterborough NHS Foundation Trust (CPFT). The data-set comprised all clozapine-treated individuals under CPFT care who died between 1 January 2009 and 31 December 2025, as identified through the Trust’s mortality review system and clozapine clinic databases. CPFT is the sole provider of secondary mental healthcare for a catchment area serving approximately one million residents across the East of England, encompassing urban, suburban and rural regions. In the UK, clozapine prescribing is subject to mandatory haematological monitoring under national regulatory requirements, delivered through approved clozapine patient-monitoring systems overseen by the Medicines and Healthcare products Regulatory Agency. In addition, clozapine initiation and ongoing treatment are overseen by specialist mental health services, with regular review by consultant psychiatrists in accordance with national guidance from the National Institute for Health and Care Excellence and local National Health Service (NHS) governance frameworks. Within this context, CPFT operates specialised clozapine clinics that provide structured clinical follow-up alongside haematological and physical health monitoring.^14^ Given these structures, it is unlikely that any clozapine-treated patient within the catchment area would not have been captured in this data-set. The treated population varied between 389 and 467 individuals across the study period, with an additional subgroup of patients moving in and out of clozapine treatment over time (e.g. during rechallenges following adverse effects, periods of non-adherence or admissions outside the Trust). Mortality rates were therefore estimated by using a representative long-term average of approximately 410 patients, on the grounds that a stable smoothed denominator provides a more realistic and reproducible approximation of the at-risk population than an artificially precise person-time figure. This is acknowledged as a source of imprecision in the Limitations section.

This study was conducted as part of an internal mortality review within CPFT. In accordance with UK Health Research Authority guidance, formal NHS Research Ethics Committee approval was not required. All data were anonymised before analysis, and the project was registered within the Trust’s clinical governance framework. It was conducted and reported in accordance with the Strengthening the Reporting of Observational Studies in Epidemiology (STROBE) guidelines for observational studies.^21^

### Identification and classification of death

**Fig. 1:**
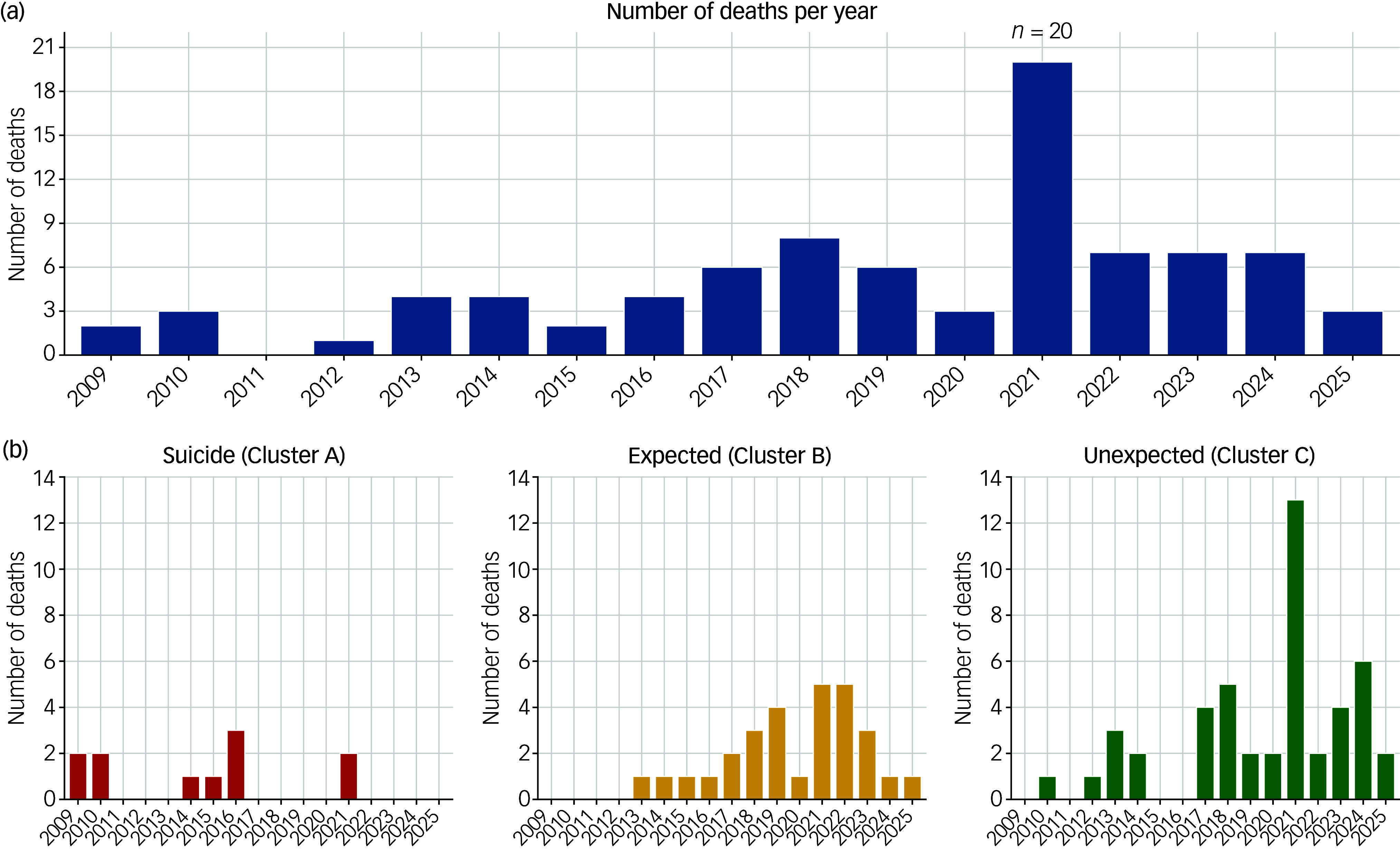
Fig. 1 long description. Total and cluster number of deaths per year.

Deceased patients were identified through multiple sources, including the CPFT Clozapine Clinic database, two manufacturer registries – the Denzapine Monitoring Service (until February 2024) and the Zaponex Treatment Access System (from February 2024) – and CPFT electronic medical records, as part of an internal mortality review audit. All deaths reported correspond to unique individuals; no patient is counted more than once. Annual counts in Fig. 1 therefore reflect the year of death of each unique deceased individual. Classification was informed by review of available clinical and mortality records, with cases assigned to one of three predefined mortality clusters, using the framework described by Rose et al.^11^

Cluster A (suicide) included intentional self-inflicted deaths, such as overdose, hanging or mixed drug toxicity. Cluster B (expected) comprised deaths attributable to chronic or progressive medical conditions, representing the natural course of long-standing physical illness. Cluster C (unexpected) encompassed sudden, non-intentional deaths occurring in the absence of a known life-limiting condition, including (a) dose-related adverse effects such as seizures, arrhythmias or gastrointestinal obstruction; (b) idiosyncratic or immune-mediated reactions, including agranulocytosis or myocarditis; (c) acute complications occurring in the context of chronic cardiometabolic disease and (d) deaths in which no definitive cause could be established despite post-mortem examination or detailed clinical review. These latter cases were classified as ‘unknown’.

For cases predating full electronic record availability, archival paper case notes were reviewed, and coroner’s inquest reports were obtained through routine Trust governance and mortality review processes, where available, to clarify the cause and circumstances of death.

Additional variables extracted for analysis included demographic characteristics (gender and age), smoking status, clinical information (attendance at mandatory annual physical health checks) and treatment-related variables, including the prescribed daily dose of clozapine. High-dose exposure was defined as a prescribed daily clozapine dose of 500 mg or higher, consistent with the threshold used in our previous catchment-area study,^11^ to preserve direct comparability between the present 17-year extension and the original 10-year cohort. We acknowledge that any threshold defined on absolute daily dose is necessarily somewhat arbitrary, as plasma concentrations achieved at a given dose vary with gender, smoking status, age and concomitant medication; plasma concentration-based exposure measures were not consistently available across the study period. Denominators in tables reflect the number of individuals with available data for each variable.

### Physical health-monitoring data

Attendance at the mandatory annual physical health check refers to the primary care assessment delivered by general practitioners under the NHS Quality and Outcomes Framework for patients on the Severe Mental Illness register, and is distinct from the routine clinical and haematological monitoring delivered within specialist clozapine clinics under CPFT. Attendance was examined across the entire clozapine-treated population under CPFT care during the study period, as a proxy indicator of continuity of physical health monitoring, and was operationalised as the most recent recorded general practitioner appointment within the preceding 12 months of the assessment date for each calendar year.

### Clozapine comparison cohort

The Clozapine Clinical and Research Database (www.schizophrenias.group/database) was used as the source of the comparison cohort. The database contains pseudonymised structured clinical assessments for approximately 250 patients treated with clozapine since 2013, comprising around 4000 recorded clinical contacts. From within this database, the analytical comparison cohort was defined as those clozapine-treated individuals who were receiving clozapine in 2019 and who remained alive through to 31 December 2025 (*n* = 156). Cases were defined as the 47 clozapine-treated individuals who died from non-intentional unexpected causes (cluster C) at any point during 2009–2025. Baseline demographic and clinical characteristics were compared at a fixed reference time point (2019) for both groups, where data were available. The 2019 reference point was chosen for both methodological and pragmatic reasons: our previous catchment area study^11^ used a baseline anchored near the start of its 2008–2018 observation window, but for the present 17-year analysis, a baseline drawn from 2008 or 2009 would have been temporally distant from many of the deaths under analysis. We therefore selected an approximate mid-period reference point (2019), which had the additional practical advantage of representing the most complete pre-pandemic snapshot of structured clinical assessments within the database. Sociodemographic variables were extracted for baseline comparisons. Uptake of the mandatory annual primary care physical health check was also examined.

### Statistical analysis

The analyses were structured around the four study aims set out in the Introduction: (a) characterisation of the specific causes of death and assignment of each death to one of the three predefined mortality clusters (suicide, expected and non-intentional unexpected), (b) cross-cluster comparison of demographic and clinical characteristics within the deceased cohort, (c) baseline comparison of unexpected death cases (cluster C) with the clozapine-treated comparison cohort alive in 2019 and (d) examination of temporal patterns in mortality and in attendance at the annual primary care physical health check across the study period.

Descriptive statistics were used to summarise demographic and clinical characteristics of the cohort. Continuous variables are presented as mean and standard deviation, and categorical variables as counts and percentages. Comparisons across mortality clusters were performed using Welch’s analysis of variance for continuous variables and Pearson’s chi-squared or Fisher’s exact test for categorical variables. Baseline comparisons between cluster C cases and the comparison cohort used Welch’s *t*-test for continuous variables and chi-squared or Fisher’s exact tests for categorical variables. Temporal patterns in selected variables, including attendance at mandatory annual physical health checks, were examined with chi-squared tests, including tests for linear trend where appropriate. All statistical tests were two-tailed, and *p* < 0.05 was considered statistically significant. Statistical analyses were performed with IBM SPSS Statistics v29.0 for macOS (IBM, New York, USA; www.ibm.com/spss) and JASP v3.0 for macOS (University of Amsterdam, The Netherlands; jasp-stats.org).

## Results

Throughout this section, percentages of deaths within mortality categories are expressed using the total of 87 deceased individuals as the denominator. Estimated annual mortality rates use the average clozapine-treated caseload under CPFT care during the study period (approximately *n* = 410) as the denominator, as described in the Method.

### Cause-of-death analysis

A total of 87 clozapine-treated patients were identified as deceased between 2009 and 2025 (see Fig. 1(a)). Annual mortality counts showed modest year-to-year variation across most of the study period, with an increase in 2018 and 2024 and a peak in 2021 (*n* = 20). No deaths were recorded in 2011. Overall, the number of deaths per year ranged from 0 to 20, with all but one year reporting fewer than eight cases.

Among the total sample, 11 deaths (12.6%) were classified as suicides (cluster A), 29 (33.3%) as expected deaths (cluster B) and 47 (54.0%) as non-intentional unexpected deaths (cluster C). As illustrated in Fig. 1(a), mortality showed an uneven temporal distribution with intermittent fluctuations and a pronounced surge in 2021. The lower panel (Fig. 1(b)) presents annual deaths stratified by cluster.

In addition to cluster classification, each case was reviewed individually to determine the specific cause of death recorded in mortality reviews or clinical documentation. A clearly identifiable primary cause was documented in 75 cases (86.2%), whereas no definitive cause could be established in 12 cases (13.8%).

Across the entire cohort, malignancy was the most frequently recorded cause of death (21/87, 24.1%). The most frequent causes were lung cancer (*n* = 5) and breast cancer (*n* = 4), followed by pancreatic (*n* = 3) and oesophageal cancer (*n* = 3). Single cases were recorded for brain, kidney and colon cancer (*n* = 1 each), as well as acute myeloid leukaemia (*n* = 1) and hepatocellular carcinoma (*n* = 1). In one case, the presence of cancer was documented, but the specific primary site could not be determined. Malignant disease was followed by cardiovascular disease (13/87, 14.9%) and respiratory or infective causes (9/87, 10.3%), including pneumonia.

Among individuals with documented treatment dates (*n* = 73), the median duration of clozapine treatment at the time of death was 14.0 years (interquartile range 8.6–17.4).

### Comparisons across mortality clusters

Comparisons across mortality clusters are summarised in Table 1. The proportion of male patients did not differ significantly between clusters (*χ* ^2^(2) = 2.18, *p* = 0.335). Age at death differed significantly across clusters (*F*(2,84) = 5.67, *p* = 0.005). *Post hoc* Fisher’s least significant difference analyses showed that patients in both the expected death and unexpected death clusters were significantly older than those who died by suicide, whereas no significant age difference was observed between expected and unexpected deaths. Smoking prevalence varied numerically across clusters, with the highest proportion observed in the unexpected death group; however, these differences did not reach statistical significance (*χ* ^2^ = 1.59, *p* = 0.453). Mean daily clozapine dose did not differ significantly across clusters (*F*(2,77) = 2.88, *p* = 0.062), although numerically higher doses were observed in the suicide and unexpected death groups compared with the expected death group. Similarly, the proportion of patients prescribed high-dose clozapine (≥500 mg/day) did not differ significantly across mortality clusters (*χ* ^2^(2) = 3.63, *p* = 0.163).

**Table 1:** Demographic and clinical characteristics of clozapine-treated patients who died between 2009 and 2025, stratified by mortality cluster Table 1 long description.

Variable	Suicide (cluster A)	Expected (cluster B)	Unexpected (cluster C)	*p*-value
Total	11	29	47	—
Male, *n* (%)	8 (72.7)	17 (58.6)	35 (74.5)	0.335
Age at death, years, mean (s.d.)	45.8 (7.8)	60.4 (11.8)	55.7 (13.4)	0.005
Smoker, *n* (%)	4 (57.1)	11 (61.1)	27 (75.0)	0.453
Clozapine dose (mg/day), mean (s.d.)	427.5 (120.4)	295.5 (155.3)	368.3 (170.3)	0.062

Data are presented as *n* (%) or mean (s.d.). Comparisons across mortality clusters were performed using Pearson’s chi-squared test for categorical variables and Welch’s analysis of variance for continuous variables. Smoking status was available for 61 individuals; clozapine dose data were available for 80 individuals.

### Comparison with the 2019 clozapine-treated cohort

At the 2019 reference time point, clozapine-treated patients who died from non-intentional unexpected causes during follow-up (2009–2025) were significantly older than those receiving clozapine in 2019 who remained alive through 2025 (comparison cohort; mean age 55.0 (s.d. 14.2) *v*. 48.6 (s.d. 10.5) years; *p* = 0.006) (Table 2). The proportion of male patients did not differ between groups (74.5 *v*. 78.8%; *p* = 0.526). Current smoking was markedly more prevalent among those who went on to die unexpectedly (75.0 *v*. 34.6%; *p* < 0.001). Mean clozapine dose in 2019 was higher among unexpected deaths, although this difference did not reach statistical significance (368.3 (s.d. 170.3) *v*. 315.3 (s.d. 145.2) mg; *p* = 0.062). The proportion of patients prescribed high-dose clozapine did not differ significantly between cases and the comparison cohort (21.3 *v*. 15.4%; *χ* ^2^(1) = 1.16, *p* = 0.281).

**Table 2:** Comparison of baseline demographic and clinical characteristics between cluster C cases (clozapine-treated patients who subsequently died from non-intentional unexpected causes between 2009 and 2025) and the comparison cohort (clozapine-treated patients receiving clozapine in 2019 who remained alive through 31 December 2025) Table 2 long description.

Variable	Comparison cohort (*n* = 156)	Cluster C cases (*n* = 47)	*p*-value
Male gender, *n* (%)	123 (78.8)	35 (74.5)	0.526
Age in 2019, years, mean (s.d.)	48.6 (10.5)	55.0 (14.2)	0.006
Smoker, *n* (%)	54 (34.6)	27 (75.0)	<0.001
Clozapine dose (mg), mean (s.d.)	315.3 (145.2)	368.3 (170.3)	0.062
High-dose clozapine (≥500 mg/day), *n* (%)	24 (15.4)	10 (21.3)	0.281

Data are presented as *n* (%) or mean (s.d.). Comparisons used Pearson’s chi-squared test for categorical variables and independent-samples *t*-tests for continuous variables. Smoking status was available for all members of the comparison cohort and for 36 cases. Clozapine dose data were available for all members of the comparison cohort and for 45 cases.

### Mortality patterns

#### COVID-19 pandemic period

Four cases (4.6%) across the entire cohort explicitly documented COVID-19 as a direct or contributing cause of death, and only two of these occurred in the absence of major concomitant medical conditions. COVID-19 was associated with one death in 2020 and three deaths in 2021. Although the proportion of COVID-19-related deaths was higher in 2021, these cases represented a small minority of all deaths recorded that year (*n* = 20), and the elevated annual mortality persisted after exclusion of COVID-19-related cases.

#### Physical health monitoring in primary care over time

**Fig. 2:**
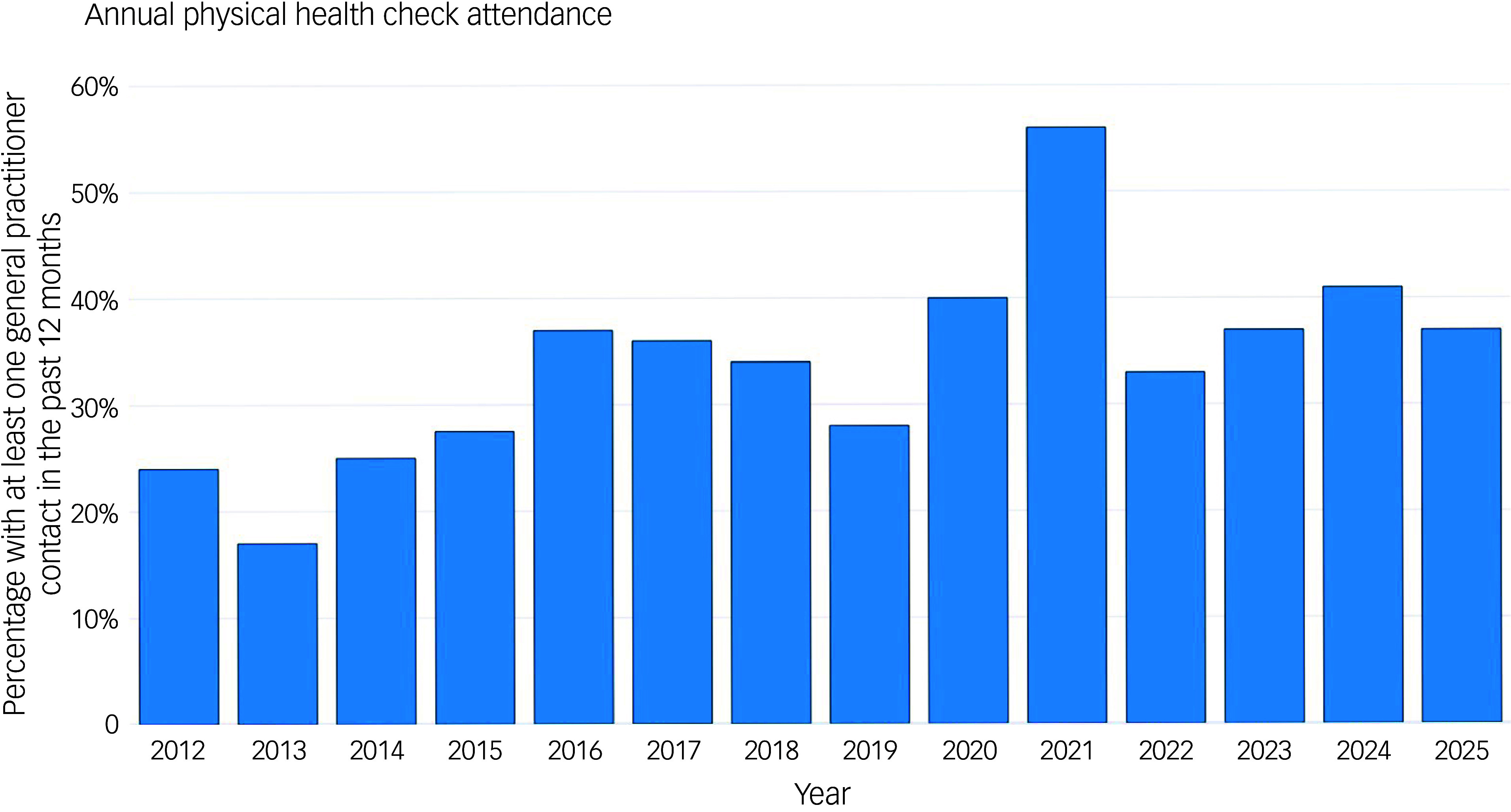
Annual percentage of clozapine-treated patients under CPFT care with a documented primary care contact within the preceding 12 months, used as a proxy for uptake of the mandatory annual physical health check, by calendar year (2012–2025). Numerator: number of clozapine-treated patients with at least one recorded general practitioner contact within the previous 12 months of the reference date. Denominator: total number of clozapine-treated patients under CPFT care during that calendar year. The measure does not capture the formal completion or quality of the annual physical health check; limitations are described in the Limitations section. CPFT, Cambridgeshire and Peterborough NHS Foundation Trust.

Attendance at mandatory annual physical health checks was examined as a proxy indicator of continuity of physical health monitoring over time. As shown in Fig. 2, attendance rates varied substantially across the study period, with a marked change around 2021. Before the COVID-19 pandemic, annual physical health check coverage ranged between 16.7 and 36.7% (2012–2019). In contrast, higher but more variable attendance rates were observed in the post-pandemic period, ranging from 32.5 to 55.5% between 2020 and 2025. Overall differences in attendance across years were statistically significant (*χ* ^2^ = 117, d.f. = 13, *p* < 0.001), with evidence of a linear association over time (*χ* ^2^ = 42.86, *p* < 0.001).

## Discussion

This study provides a long-term detailed overview of mortality among clozapine-treated patients within a defined UK catchment area, characterising predominant causes of death and temporal patterns over a 17-year period. Deaths were grouped into three predefined clusters (suicide, expected and non-intentional unexpected), providing a structured descriptive framework for examining the heterogeneous clinical contexts in which death occurred. Even among patients classified within the expected death cluster, the mean age at death was approximately 60 years, underscoring the substantially reduced life expectancy observed in this population compared with the general population, which, according to the most recent Office for National Statistics data (2020–2022), is approximately 80.0 years for males and 83.7 years for females in the East of England – the region served by CPFT.^22^ Notably, in a separate baseline comparison with a clozapine-treated comparison cohort alive in 2019, current smoking was markedly more prevalent among individuals who subsequently died from non-intentional unexpected causes. Taken together, examining mortality patterns through a cluster-based framework, alongside a baseline case–comparison analysis between unexpected death cases (cluster C) and a clozapine-treated comparison cohort alive in 2019, may offer a useful descriptive approach for understanding the diverse pathways through which fatal outcomes arise in clozapine-treated populations.

### Cluster-specific mortality patterns

Suicide (cluster A) constituted the smallest group, comprising 11 out of 87 deaths (12.6%). When considered against the estimated number of clozapine-treated individuals within the catchment (around 410 at any given time), this corresponds to an annual mortality of 0.15%. Eleven suicides over 17 years among an average treated population of approximately 410 patients corresponds to an estimated rate of approximately 158 per 100 000 person-years. UK data from a large secondary mental healthcare cohort show a substantial overall mortality reduction among clozapine-treated individuals with serious mental illness (adjusted hazard ratio 0.4, 95% CI 0.2–0.7),^23^ although suicide-specific rates were not stratified by clozapine status in that study. Direct head-to-head comparisons of clozapine versus non-clozapine treatment in large nationwide register studies indicate a particularly marked reduction in suicide-specific mortality with clozapine: the Finnish FIN20 cohort reported an adjusted hazard ratio of 0.21 (95% CI 0.15–0.29) for suicide-specific mortality with clozapine versus no antipsychotic exposure,^2^ and a Taiwanese nationwide study reported a significant, dose-dependent reduction in suicide mortality with clozapine relative to matched non-clozapine controls.^4^ The rate observed in the present cohort sits at the lower end of expected values for patients with schizophrenia spectrum disorder under specialist mental healthcare and is broadly comparable with that observed in our previous catchment area cohort over 2008–2018,^11^ consistent with the well-established suicide-protective effect of clozapine relative to other antipsychotics.

Expected deaths (cluster B) accounted for 29 out of 87 cases (33.3%) and were predominantly associated with chronic or progressive medical illness. Recent population-based evidence has reported a rare but increased incidence of haematological malignancies among clozapine-treated patients,^24^ described as a composite outcome rather than specific diagnostic subtypes. In the present cohort, malignancy was indeed the most frequently recorded cause of death (21/87, 24.1%), but was predominantly attributable to solid tumours – particularly lung, breast, pancreatic and oesophageal cancers – whereas only a single death was attributable to a haematological malignancy (acute myeloid leukaemia). Of relevance, several of the solid tumours observed are malignancies associated with tobacco.^25^ Other causes within this cluster reflected the cumulative burden of long-term physical comorbidity – such as advanced cardiometabolic conditions, chronic respiratory disease and multi-organ decline – which are frequently observed in ageing individuals with long-standing psychotic disorders.^1^

Unexpected deaths (cluster C) represented the largest proportion of the cohort (47 out of 87, 54%) and encompassed a heterogeneous set of clinical presentations. Age at death in this group was relatively low, with a mean of 55.7 years (s.d. 13.4). Approximately half of the cases in this cluster may have involved acute decline – including abrupt cardiac or respiratory compromise, gastrointestinal obstruction or perforation, infectious complications or metabolic disturbances – whereas others were attributed to longer-term cardiometabolic disease such as ischaemic heart disease. Unclassified causes of death were observed in 12 cases (13.8%), reflecting sudden or unwitnessed fatalities where available clinical or post-mortem information was insufficient for definitive classification, as is often the case in presumed arrhythmic events. Similar patterns have been reported previously, including in CPFT audits^11^ and pharmacovigilance-informed reviews, where acute and potentially preventable events constituted a substantial proportion of deaths. These observations are consistent with recent global pharmacovigilance data, in which fatal pneumonia represents a substantially greater contribution to mortality than agranulocytosis among clozapine-treated patients.^15^

In aggregate, the three clusters describe a range of clinical contexts in which death occurred among clozapine-treated patients without implying that these clusters represent mutually exclusive pathways of risk.

### Clinical characteristics associated with unexpected deaths

In the baseline comparison with the comparison cohort alive in 2019, current smoking was markedly more prevalent among individuals who subsequently died from non-intentional unexpected causes (75 *v*. 34.5%), whereas smoking prevalence did not differ significantly across mortality clusters within the full cohort. Mean clozapine dose at the reference time point was also numerically higher among unexpected deaths, although this difference did not reach statistical significance and may partly reflect higher dose requirements among individuals who smoke, given induction of clozapine metabolism. Patients who died from unexpected causes were also older at the reference time point. The high prevalence of smoking observed in this subgroup is consistent with population-based evidence indicating that approximately 40–60% of people with severe mental illness smoke, compared with around 14% of the general adult population in the UK,^26^ and that tobacco use contributes substantially to excess physical morbidity and premature mortality in this population.^27,28^

Across cluster-based comparisons within the full clozapine-treated cohort, age was the principal factor differentiating mortality groups overall. Notably, even individuals classified within the expected death cluster died at a mean age of approximately 60 years, substantially lower than life expectancy in the general UK population. By contrast, gender distribution, mean prescribed clozapine dose, smoking status and exposure to high-dose clozapine did not differ significantly across mortality clusters, suggesting that dose-related factors alone do not account for the observed heterogeneity in fatal outcomes.

### Temporal variation in mortality and contextual factors

Mortality varied across the 17-year period, with notable increases in 2018 (*n* = 8) and 2024 (*n* = 6), and a pronounced maximum in 2021 (*n* = 20). These peaks coincided with periods characterised by broader environmental and systemic stressors, although no direct causal attribution can be made.

Of note, the highest number of deaths occurred in 2021, rather than during the initial phase of the COVID-19 pandemic in March 2020. This increase coincided with the second and third pandemic waves; however, only four deaths in the cohort involved SARS-CoV-2 infection, and in just two cases was COVID-19 recorded in the absence of another major competing medical cause. Although systemic inflammation during acute infection is known to inhibit CYP1A2 metabolism, with the potential to increase clozapine plasma concentrations,^29^ the excess mortality observed in 2021 is unlikely to be accounted for by these cases alone.

Indirect consequences of the pandemic – such as disruption to routine physical health monitoring, reduced access to services, delays in help-seeking^30^ and exacerbation of pre-existing medical comorbidity – may have contributed to increased vulnerability during this period.^31,32^ From a broader clinical and social perspective, individuals with treatment-resistant psychotic disorders often experience impaired insight, limited support networks and reduced engagement with routine healthcare; factors that may further compromise timely assessment and continuity of care during periods of system-wide disruption.^31^ In this context, the observed temporal variation in attendance at mandatory annual physical health checks (Fig. 2) may reflect fluctuations in access to and continuity of routine physical health monitoring during and following the pandemic period.

### Clinical implications

Overall, the findings suggest that vulnerability to mortality among clozapine-treated patients reflects a combination of clinical, physical and behavioural factors rather than medication dose alone. From a clinical perspective, these results support comprehensive surveillance integrating regular assessment of clozapine-related adverse effects, standardised physical health review and longitudinal follow-up. In this context, the use of validated tools such as the Glasgow Antipsychotic Side-Effect Scale for Clozapine^33^ may aid systematic identification of emerging adverse effects.

Beyond direct pharmacological mechanisms, the structured nature of clozapine treatment – including frequent clinical contact, mandatory haematological monitoring and regular physical health review – may itself contribute to its observed mortality advantage. Such contact provides repeated opportunities for early identification of comorbidity, for supporting adherence to lipid-lowering and antihypertensive therapy, and for engagement with smoking-cessation and substance-use interventions. The relative reduction in tobacco and illicit-substance use described in some clozapine cohorts may further contribute to this effect.

There is an emerging international consensus that the intensity of long-term clozapine haematological monitoring may be safely reduced for stable patients.^34,35^ Although such liberalisation may improve quality of life and reduce treatment burden, our findings underscore that mandatory monitoring also functions as a structured framework for regular clinical contact, indirect physical health oversight and the identification of modifiable risk factors. Any reform of monitoring requirements should therefore be paired with deliberate redesign of clozapine services to preserve continuity of care and to safeguard the cardiometabolic and infectious disease surveillance that this population requires.^36^

Reductions or disruptions in this process may indirectly increase vulnerability by limiting opportunities for close follow-up, early detection of emerging physical or treatment-related complications, and identification of modifiable risk factors, including smoking, as well as access to evidence-based smoking-cessation programmes, which remain underutilised among people with severe mental illness.^27,37^

### Limitations

This study has several limitations inherent to its retrospective design. The determination of cause of death relied on the completeness and accuracy of clinical documentation, coroner’s reports and historical records, which may have resulted in misclassification in some cases. The analyses presented are descriptive and based on between-group comparisons; no formal survival or time-to-event modelling was undertaken. Consistent clozapine initiation dates could not be retrieved for some cases predating full electronic record availability, which would have been required for time-to-event analysis. The relatively small size of subgroups – particularly the suicide cluster – restricted statistical power to detect between-group differences.

The comparison cohort comprised individuals who were receiving clozapine in 2019 and who remained alive through to the end of the study period. By design, this introduces survivorship and immortal time bias and may distort observed associations – particularly for age and smoking status – toward apparent risk among unexpected death cases. The 2019 reference year was nevertheless chosen because it represents the most complete pre-pandemic baseline available within the database and provided the largest comparison cohort with sufficient subsequent follow-up. Comparisons should therefore be interpreted as descriptive rather than causal.

Mortality rates were calculated using an average estimated clozapine-treated caseload as the denominator, despite year-to-year variation in the size of the treated population. This approach may have introduced imprecision in rate estimates.

Attendance at mandatory annual physical health checks was operationalised by using recorded timing of general practitioner contact and used as a proxy for continuity of physical health monitoring. However, general practitioner contact does not necessarily correspond to completion of a formal annual physical health check and may reflect heterogeneous types of encounters. This measure is therefore susceptible to recall bias, variability in recording practices and missing or uncertain dates, and does not capture the quality, depth or clinical impact of assessments, nor informal or unrecorded healthcare contacts. As such, its face validity as a measure of annual physical health check uptake is limited.

Finally, the findings reflect the characteristics and healthcare structures of a single catchment area and may not be fully generalisable to other settings.

In conclusion, mortality among clozapine-treated patients in this 17-year cohort arose across diverse clinical contexts, with more than half of deaths classified as non-intentional and unexpected. Malignancy, cardiovascular disease and infective causes predominated, and current smoking was markedly more prevalent among individuals who later died unexpectedly. These findings underscore the importance of sustained physical health monitoring, focused attention to modifiable risks – particularly smoking – and continuity of specialist and primary care input for clozapine-treated patients, especially during periods of system-wide disruption.

## Data Availability

The data that support the findings of this study are available on request from the corresponding author, E.F.-E. The data are not publicly available due to their containing information that could compromise the privacy of research participants.

## Fig. 1 Long description

Panel A: A vertical bar graph shows the number of deaths per year from 2009 to 2025. The horizontal axis represents the years, and the vertical axis represents the number of deaths. The graph shows a significant peak in deaths in 2021 with 20 deaths. Panel B: Three separate vertical bar graphs show the number of deaths by cause: Suicide (Cluster A), Expected (Cluster B), and Unexpected (Cluster C). Each graph has the same horizontal axis representing the years from 2009 to 2025 and the vertical axis representing the number of deaths. The Suicide (Cluster A) graph shows a few deaths scattered over the years. The Expected (Cluster B) graph shows a gradual increase in deaths over the years. The Unexpected (Cluster C) graph shows a significant peak in deaths in 2021 and a gradual increase in subsequent years.

Navigate back to Fig. 1.

## Table 1 Long description

The table compares demographic and clinical characteristics of clozapine-treated patients who died between 2009 and 2025, stratified by mortality cluster. It has four rows and five columns. The columns are labeled Variable, Suicide (cluster A), Expected (cluster B), Unexpected (cluster C), and p-value. The rows are labeled Total, Male, n (%), Age at death, years, mean (s.d.), Smoker, n (%), and Clozapine dose (mg/day), mean (s.d.). Row 1: Total, 11, 29, 47, p-value. Row 2: Male, n (%), 8 (72.7 percent), 17 (58.6 percent), 35 (74.5 percent), 0.335. Row 3: Age at death, years, mean (s.d.), 45.8 (7.8), 60.4 (11.8), 55.7 (13.4), 0.005. Row 4: Smoker, n (%), 4 (57.1 percent), 11 (61.1 percent), 27 (75.0 percent), 0.453. Row 5: Clozapine dose (mg/day), mean (s.d.), 427.5 (120.4), 295.5 (155.3), 368.3 (170.3), 0.062.

Navigate back to Table 1.

## Table 2 Long description

The table compares baseline demographic and clinical characteristics between cluster C cases (clozapine-treated patients who subsequently died from non-intentional unexpected causes between 2009 and 2025) and the comparison cohort (clozapine-treated patients receiving clozapine in 2019 who remained alive through 31 December 2025). The table has 6 rows and 4 columns. Column headers are Variable, Comparison cohort (n = 156), Cluster C cases (n = 47), and p-value. Row 1: Male gender, n (percent), 123 (78.8), 35 (74.5), 0.526. Row 2: Age in 2019, years, mean (s.d.), 48.6 (10.5), 55.0 (14.2), 0.006. Row 3: Smoker, n (percent), 54 (34.6), 27 (75.0), less than 0.001. Row 4: Clozapine dose (mg), mean (s.d.), 315.3 (145.2), 368.3 (170.3), 0.062. Row 5: High-dose clozapine (greater than or equal to 500 mg/day), n (percent), 24 (15.4), 10 (21.3), 0.281.

Navigate back to Table 2.
